# Negative Roles of Strigolactone-Related SMXL6, 7 and 8 Proteins in Drought Resistance in *Arabidopsis*

**DOI:** 10.3390/biom10040607

**Published:** 2020-04-14

**Authors:** Weiqiang Li, Kien Huu Nguyen, Cuong Duy Tran, Yasuko Watanabe, Chunjie Tian, Xiaojian Yin, Kun Li, Yong Yang, Jinggong Guo, Yuchen Miao, Shinjiro Yamaguchi, Lam-Son Phan Tran

**Affiliations:** 1 Institute of Plant Stress Biology, State Key Laboratory of Cotton Biology, Department of Biology, Henan University, 85 Minglun Street, Kaifeng 475001, China; weiqiangli@henu.edu.cn or likun@henu.edu.cn (K.L.); 104752160097@vip.henu.edu.cn (Y.Y.); jgguo@henu.edu.cn (J.G.); miaoych@henu.edu.cn (Y.M.); 2 Stress Adaptation Research Unit, RIKEN Center for Sustainable Resource Science, 1-7-22, Suehiro-cho, Tsurumi, Yokohama 230-0045, Japan; tdcuong87@gmail.com (C.D.T.); yasuko.watanabe@riken.jp (Y.W.); 3 Agricultural Genetics Institute, Vietnam Academy of Agricultural Sciences, Pham Van Dong Str., Hanoi 100000, Vietnam; kienbio280888@gmail.com; 4 Key Laboratory of Mollisols Agroecology, Northeast Institute of Geography and Agroecology, Chinese Academy of Sciences, Changchun 130102, China; tiancj@iga.ac.cn; 5 State Key Laboratory of Natural Medicines, Department of Pharmacognosy, Institute of Pharmaceutical Science, China Pharmaceutical University, Nanjing 210009, China; xiaojian@cpu.edu.cn; 6 Institute for Chemical Research, Kyoto University, Uji, Kyoto 611-0011, Japan; shinjiro@scl.kyoto-u.ac.jp; 7 Institute of Research and Development, Duy Tan University, 03 Quang Trung, Da Nang 550000, Vietnam

**Keywords:** strigolactone, SMXL, drought resistance, abscisic acid

## Abstract

Previous investigations have shown that the SUPPRESSORS OF MAX2 1-LIKE6, 7 and 8 (SMXL6, 7 and 8) proteins redundantly repress strigolactone (SL) signaling in plant growth and development. Recently, a growing body of evidence indicated that SLs positively regulate plant drought resistance through functional analyses of genes involved in SL biosynthesis and positive regulation of SL signaling. However, the functions of the SL-signaling negative regulators SMXL6, 7 and 8 in drought resistance and the associated mechanisms remain elusive. To reveal the functions of these SMXL proteins, we analyzed the drought-resistant phenotype of the triple *smxl6*,*7*,*8* mutant plants and studied several drought resistance-related traits. Our results showed that the *smxl6*,*7*,*8* mutant plants were more resistant to drought than wild-type plants. Physiological investigations indicated that the *smxl6*,*7*,*8* mutant plants exhibited higher leaf surface temperature, reduced cuticle permeability, as well as decreases in drought-induced water loss and cell membrane damage in comparison with wild-type plants. Additionally, *smxl6*,*7*,*8* mutant plants displayed an increase in anthocyanin biosynthesis during drought, enhanced detoxification capacity and increased sensitivity to abscisic acid in cotyledon opening and growth inhibition assays. A good correlation between the expression levels of some relevant genes and the examined physiological and biochemical traits was observed. Our findings together indicate that the SMXL6, 7 and 8 act as negative regulators of drought resistance, and that disruption of these *SMXL* genes in crops may provide a novel way to improve their drought resistance.

## 1. Introduction

Strigolactones (SLs) are a new member of plant hormones, regulating plant growth and development, and plant responses to environmental stresses [1,2,3,4]. In *Arabidopsis*, SLs are biosynthesized from carotenoids through a series of enzymes, namely *Arabidopsis thaliana* DWARF27 (D27), MORE AXILLARY GROWTH 3 (MAX3), MAX4, MAX1 and LATERAL BRANCHING OXIDOREDUCTASE [4]. Even the endogenous active SLs and their biosynthetic pathway in *Arabidopsis* have not been fully identified yet, several signal transduction components have been well documented recently [4,5].

In *Arabidopsis*, the SL signaling pathway comprises of two positive regulators *Arabidopsis* D14 protein (D14) and MAX2, and three redundant negative regulators SUPPRESSOR OF MAX2 1-LIKE6, 7 and 8 (SMXL6, 7 and 8) [1,4,6,7]. The first step of SL signal transduction is the binding of SLs to the pocket of D14 protein, which is an α/β-hydrolase protein and possesses both enzyme and receptor activities [8,9,10,11]. Next, the D14 protein undergoes conformational changes and recruits MAX2, which is an F-box protein and functions as an adaptor component of a Skp1-Cullin-F-box (SCF) E3 ubiquitin ligase complex [10,12]. The newly formed D14-MAX2-SCF complex polyubiquitinates the three redundant SMXL6, 7 and 8 repressors, and triggers the degradation of these negative regulators, releasing SL-responsive genes and consequently resulting in SL-regulated phenotypes associated with shoot branching, lateral root growth, primary root growth and leaf shape, etc. [6,7,13,14]. For example, mutations of all three *SMXL6, 7* and *8* genes in *max2* and *max3* backgrounds suppressed *max2* and *max3* shoot branching, and leaf shape and lateral root phenotypes that are specifically associated with the SL signaling [6,7]. It is worth mentioning that the MAX2 protein also plays a central role in a sister pathway mediated by a D14 homolog called KARRIKIN-INSENSITIVE2 (KAI2) that receives signals from karrikins (KARs) found in smoke [2].

Recent investigations in various plant species reported positive roles of SLs in drought resistance through functional analyses of various mutants with defect in SL-biosynthetic or SL positive regulatory genes, such as the *Arabidopsis* SL-biosynthetic *max3* and *max4* and SL-signaling *max2* and *d14* mutant plants [15,16,17,18,19], and the *Lotus japonicus* and tomato (*Solanum lycopersicum*) SL-depleted transgenic plants silenced for a *MAX3* homolog, named *CAROTENOID CLEAVAGE DIOXYGENASE 7* (*CCD7*) gene [3,20,21]. Physiological and biochemical analyses of these mutants in relation to drought stress revealed that SL signaling regulates a number of drought-related mechanisms, including water transpiration, abscisic acid (ABA) responsiveness, leaf senescence, cell membrane integrity and anthocyanin biosynthesis [15,16,17,18,19,20,21]. However, the roles of the negative regulators SMXL6, 7 and 8 and associated mechanisms underlying drought resistance are still unknown. Dissection of the roles of SMXL6, 7 and 8 in the *Arabidopsis* plant response to drought will allow us to provide the whole picture of the functions of the SL signaling in drought resistance. Thus, in this study, we compared the drought resistance, and several drought-related physiological and biochemical traits between the *smxl6*,*7*,*8* mutant and wild-type (WT) plants. Our results showed that the mutations of *SMXL6*, *7* and *8* genes in *Arabidopsis* enhanced plant drought resistance of the *smxl6*,*7*,*8* mutant plants through preventing leaf water loss, enhancing ABA responsiveness, and promoting anthocyanin biosynthesis and reactive oxygen species (ROS)-scavenging activities.

## 2. Materials and Methods

### 2.1. Plant Materials and Generation of Plate-Grown Seedlings

*Arabidopsis thaliana* Col-0 ecotype was used as WT. Seeds of the *smxl6-4*,*7-3*,*8-1* (named *smxl6*,*7*,*8* hereafter) mutant in the Col-0 background were originally generated by Soundappan et al. (2015) [7], where the *smxl6-4, smxl 7-3* and *smxl8-1* are SALK_049115, WiDsLox339_C04 and SALK_025338C lines, respectively. Other T-DNA alleles used in this study were *smxl6-5* (SALK_201861C), *smxl7-4* (SALK_082032) and *smxl8-2* (SALK_126406) that were obtained from the Arabidopsis Biological Resource Center. The double mutants *smxl6-5,7-4* (named *smxl6*,*7* hereafter), *smxl7-4*,*8-2* (named *smxl7*,*8* hereafter) and *smxl6-5*,*8-2* (named *smxl6*,*8* hereafter), and the triple mutant *smxl6-5*,*7-4*,*8-2* (named *smxl6*,*7*,*8-2* hereafter) were generated by crossing the single mutants. WT and different mutant seeds were sown on germination medium (GM) agar medium, and the plates were placed at 4 °C in the dark [17]. Three days later, the plates were moved to a growth chamber (16-h-light/8-h-dark cycle, 60 μmol m^−2^ s^−1^ photon flux density, 22 °C) for two weeks to generate two-week-old plate-grown seedlings.

### 2.2. Drought Resistance Assays

To evaluate drought resistance, the ‘same tray’, ‘one pot’ and ‘weighing’ methods were used. For the ‘same tray’ method, two-week-old plate-grown WT and different mutant seedlings were transferred in pairs (30 plants/genotype) to trays (21 cm × 30 cm × 5 cm in width, length and depth) containing commercial soil (Dio Propagation Mix No. 2 for Professional, Dio Chemicals, Tokyo, Japan) as previously described [22]. After the plants were grown for an additional 7 days, water irrigation was stopped until distinguishable differences were observed between WT and investigated mutant plants. Rewatering was performed, and survived plants were counted three days after rewatering to evaluate survival rate. Pictures of plants (after inflorescences were removed) were taken after rewatering for five days and shown as representative pictures. For the ‘one pot’ system, two-week-old plate-grown seedlings were transplanted to soil confined in one small pot (7 cm × 7 cm in diameter and height). The growing, drying, rewatering and photographing processes of the ‘one pot’ system were similar to that of the ‘same tray’ method. For the ‘weighing’ method, two-week-old plate-grown seedlings were separately transplanted to the pots with the same size as used in the ‘one pot’ system. The pot weights were measured and recorded each day following a previous method [17]. After the drought treatment for 17 days, the whole shoot parts (with inflorescence) of drought-treated and well-watered control plants were collected and packed in paper bags. The bags were oven-dried at 65 °C for 48 h, and the biomass (dry weight, DW) of each shoot sample was recorded. The percentage of biomass reduction was determined according to Equation (1):

Biomass reduction (%) = ((DW of well-watered plant − DW of stressed plant) × 100)/(DW of well-watered plant)(1)

### 2.3. Relative Water Content (RWC), Electrolyte Leakage and Anthocyanin Content

Relative water content (RWC), electrolyte leakage and anthocyanin content were measured in shoots (without inflorescence) of WT and *smxl6*,*7*,*8* mutant plants during the soil-drying treatment following the published procedures [22,23]. Briefly, WT and *smxl6*,*7*,*8* mutant plants (30 plants/genotype) were grown in the same tray as previously described for the ‘same tray’ method of drought resistance assay. Plant samples were collected after withholding water for 11–15 days for determination of fresh weight (FW) at different time points. Plant samples were then immersed in distilled water at room temperature with shaking for 3 h. Then, the plant samples were taken out, water was removed from the plant surface by using tissue papers, and sample weights were measured as turgid weight (TW). Plant samples were then packed in paper bags and oven-dried at 65 °C for 48 h, and the DW of each sample was recorded. RWCs of the plant samples for both soil drying and dehydration (Section 2.4) treatments were determined using Equation (2):

RWC (%) = 100 × (FW − DW)/(TW − DW)(2)

Electrolyte leakage and anthocyanin contents were measured in each plant sample after withholding water for 11–15 days [22,23]. Soil moisture contents and relative air humidity were also determined daily during drying, following the methods previously described [22].

### 2.4. Leaf Surface Temperature, Dehydration Treatment, Toluidine Blue (TB) Staining and Chlorophyll (Chl) Leaching Assays

Two-week-old plate-grown seedlings were transplanted to soil and grown for one week under well-watered conditions (21-day-old seedlings). Leaf surface temperatures of seedlings were estimated from the seedlings grown with irrigation and without irrigation for 7 days (28-day-old seedlings) by using an infrared thermal camera system (R500EX-S; Nippon Avionics, Tokyo, Japan). For the dehydration treatment, two-week-old plate-grown seedlings were transplanted to soil and grown for 10 days under well-watered conditions (24-day-old seedlings). FWs of harvested 24-day-old seedlings (without inflorescence) were recorded after different time periods of dehydration (0.5–8 h). The TW and DW measurements, and RWC calculation were followed as previously described for the soil-drying treatment (Section 2.2 and Section 2.3). The 28-day-old well-watered seedlings were also used in TB staining and Chl leaching assays as previously described to detect cuticle defect on leaves [17,24].

### 2.5. Evaluation of ABA Responsiveness Using Cotyledon Opening and Growth Inhibition Assays

Seeds of WT and *smxl6*,*7*,*8* mutant plants were sown on GM plates supplemented with various ABA concentrations, which were then incubated in a growth chamber with the same growth conditions as described in Section 2.1. Percentages of cotyledon opening were determined according to the published method [22]. After two weeks, whole seedlings from the GM plates were harvested and FWs (six seedlings/reading) were measured. Relative FWs were determined using Equation (3):

Relative FW (%) = 100 × (FW of plants with ABA treatment/FW of plants without ABA treatment)(3)

### 2.6. In Situ Detection of ROS and Plant Response to Oxidative Stress

Superoxide (O_2_**^.^**^–^) and hydrogen peroxide (H_2_O_2_) levels were estimated by staining four-week-old WT and *smxl6*,*7*,*8* mutant plants (grown as described for TB staining; Section 2.4) with nitro blue tetrazolium (NBT) and 3,3′-diaminobenzidine (DAB), respectively, according to the previously published protocols with several minor modifications [25]. For O_2_**^.^**^–^ detection, rosette leaves of four-week-old seedlings were immersed in NBT solution (0.1% NBT, 10 mM NaN_3_, 10 mM phosphate buffer, pH 7.8), and were incubated for 2 h under room temperature and continuous light to visualize dark blue spots. For H_2_O_2_ detection, rosette leaves of four-week-old seedlings were immersed in DAB solution (0.1% DAB, 10 mM phosphate buffer, pH 7.8), and were incubated for 8 h under room temperature and continuous light to visualize brown spots. For both NBT and DAB staining treatments, the treated rosettes were bleached in 100% ethanol, and pictures were taken after the samples were transferred to water for 2 h.

To compare the oxidative stress tolerance of *smxl6*,*7*,*8* mutant and WT plants, N, N′-dimethyl-4,4′-bipyridinium dichloride (paraquat; PQ) was used as the source of O_2_**^.^**^–^ radicals [26]. Furthermore, 3-amino-1,2,4-triazole (3-AT) was also explored as an irreversible inhibitor of catalase that scavenges H_2_O_2_ and produces water and O_2_ [27]. The assays with PQ and 3-AT were conducted following the published methods with some modifications [28]. Briefly, 10-day-old plate-grown seedlings were transferred to new GM plates containing different concentrations of 3-AT (0, 15 and 30 μM) and PQ (0.5 and 1.0 μM), and grown for 11 days under the same growth conditions as described in Section 2.1. Subsequently, 21-day-old whole seedlings were harvested for measuring FWs. Relative FWs were determined using Equation (4):

Relative FW (%) = 100 × (FW of plants with 3-AT or PQ treatment/FW of plants without 3-AT or PQ treatment)(4)

### 2.7. Quantitative Reverse Transcriptase-PCR (qRT-PCR) Analysis

For gene expression analysis using qRT-PCR, two-week-old plate-grown WT and *smxl6*,*7*,*8* mutant plants were transplanted to soil and grown for 10 days. The 24-day-old shoot parts (without inflorescence) were then harvested and subjected to a dehydration treatment. The rosette leaves were collected after 0, 2 and 4 h dehydration in three biological replicates (*n* = 3). RNA purification, cDNA synthesis and qRT-PCR were carried out according to the reported procedures [29]. Primers used in the qRT-PCR analysis of examined genes, including the *UBQ10* reference gene, are listed in Table S1.

## 3. Results

### 3.1. Arabidopsis smxl6,7,8 Mutant Plants Exhibit Enhanced Drought Resistance

**Figure 1:**
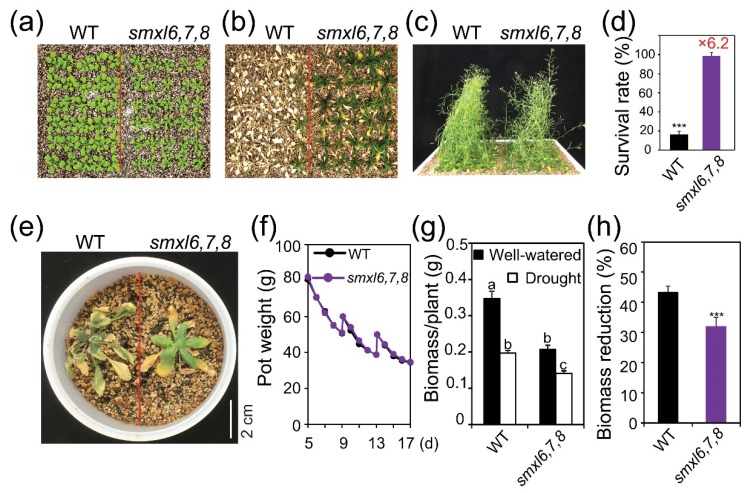
Enhanced drought resistance of *smxl6*,*7*,*8* mutant plants. Comparisons of *smxl6*,*7*,*8* mutant and wild-type (WT) plants were performed using the ‘same tray’ (**a**–**d**), ‘one pot’ (**e**) and ‘weighing’ (**f**–**h**) methods. (**a**) WT and *smxl6*,*7*,*8* mutant plants were grown for 21 days under well-watered conditions in a tray. (**b**) Water was withheld to observe distinguishable differences between the two genotypes. After 15 days of water withholding, rewatering was conducted. A picture was taken five days after rewatering, and after inflorescences were removed. (**c**) Control well-watered plants were grown in parallel with the drought resistance assay. (**d**) Means and standard errors (SEs) of three independent experiments (*n* = 3, 30 plants/genotype/experiment) were used to estimate the survival rates of investigated WT and *smxl6*,*7*,*8* mutant plants. Red number above the error bar indicates the fold-change in survival rate of *smxl6,7,8* mutant over the WT. (**e**) WT and *smxl6,7,8* plants were grown side-by-side in a small pot. Water was withheld to observe distinguishable differences between the two genotypes. After 15 days of water withholding, rewatering was conducted. Picture was taken five days after rewatering, and after inflorescences were removed. (**f**) Pot weights during the soil-drying process of the ‘weighing’ method. Data are means and SEs (*n* = 12 pots/genotype). (**g**) Shoot dry weights of WT and *smxl6,7,8* mutant plants were measured at day 17th of the well-watered or drought treatment. (**h**) Shoot biomass reduction percentages of WT and *smxl6*,*7*,*8* mutant plants at day 17th of the ‘weighing’ assay. Data are means and SEs (*n* = 12 plants/genotype). Letters above the error bars indicate significant differences (Tukey’s honest significant difference test; *p* < 0.05). Asterisks show significant differences between the two genotypes (Student’s *t*-test; *** *p* < 0.001).

To compare drought resistance between WT and *smxl6*,*7*,*8* mutant plants, we first evaluated the survival rates of the two genotypes using the ‘same tray’ method (Figure 1a–d). Our data revealed a significantly higher survival rate (6.2-fold) of *smxl6*,*7*,*8* mutant than that of WT plants (Figure 1b,d). This finding was supported by the 8.6-fold higher survival rate of another triple mutant line, namely the *smxl6*,*7*,*8-2*, over the WT plants in an independent soil-drying assay using the ‘same tray’ method (Figure S1). Further detailed investigations of various double and single mutant combinations in the ‘same tray’ assays revealed that the *smxl6*,*7*, *smxl6*,*8* and *smxl7*,*8* double mutants displayed 2.0-fold, 1.4-fold and 1.4-fold higher survival rate, respectively, than WT, while the *smxl6*, *smxl7* and *smxl8* single mutant and WT plants showed comparable survival rates (Figure S1). These results collectively indicated that the three SMXL6, 7 and 8 proteins acted as negative regulators of drought resistance with functional redundancy in *Arabidopsis* plants.

Higher drought resistance was also noted with *smxl6*,*7*,*8* mutant versus WT plants by using the ‘one pot’ system (Figure 1e). Although the ‘same tray’ and ‘one pot’ systems were explored to ensure valuable comparison of the two genotypes in terms of drought resistance, since these two genotypes showed remarkable size differences (Figure 1a) [30], the ‘weighing’ method (Figure 1f) was also used to strengthen the obtained results. We noted that the shoot biomass of *smxl6*,*7*,*8* mutant was lower than that of WT plants under normal growth conditions (Figure 1g). However, the biomass reduction percentage of *smxl6*,*7*,*8* mutant was lower than that of WT plants, when the two genotypes were subjected to the growth conditions of similar soil water contents (Figure 1f,h), clearly indicating the enhanced drought resistance of *smxl6*,*7*,*8* mutant versus WT plants. Taken together, the results of drought resistance assays convincingly demonstrated that the SMXL6, 7 and 8 proteins negatively and redundantly regulate drought resistance in *Arabidopsis* plants.

Next, we were curious about the responses of *SMXL6, 7* and *8* genes to water-deficit conditions. Thus, we examined the expression of these three genes in WT plants exposed to dehydration using qRT-PCR. As shown in Figure S2, dehydration treatment triggered down-regulation of the *SMXL6* and *SMXL7* genes that exhibited significantly decreased expression levels after both 2 h and 4 h of dehydration. *SMXL8* gene showed a slight reduction at 2 h, and then a weak induction at 4 h of dehydration. These data further strengthened the involvement of *SMXL6*, *7* and *8* genes in regulation of plant response to water stress, and suggested that dehydration might trigger down-regulation of these *SMXL* genes, at least at earlier time points, resulting in adaptive responses of *Arabidopsis* plants to water-limited conditions.

### 3.2. Arabidopsis smxl6,7,8 Mutant Plants Show Reduced Water Loss and Electrolyte Leakage, and Increased Anthocyanin Content during Drought

**Figure 2:**
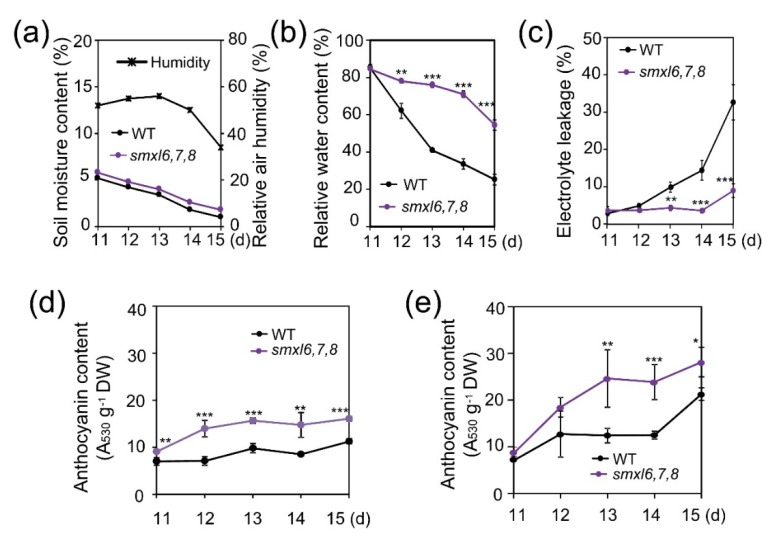
Relative water contents, electrolyte leakage percentages and anthocyanin contents obtained from *smxl6*,*7*,*8* mutant and wild-type (WT) plants during the soil-drying experiment using the ‘same tray’ method. (**a**) Soil moisture content and relative air humidity during the soil-drying. (**b**,**c**) Relative water contents (**b**) and electrolyte leakage percentages (**c**) of the two genotypes during the soil-drying. (**d**,**e**) Anthocyanin contents in the two genotypes under well-watered (**d**) and progressive soil-drying (**e**). Data are means and SEs (*n* = 4 plants/genotype). Asterisks show significant differences between the *smxl6*,*7*,*8* mutant and WT plants at each time point (Student’s *t*-test; * *p* < 0.05, ** *p* < 0.01 and *** *p* < 0.001).

We next examined several physiological and biochemical traits that might contribute to the enhanced drought resistance of the *smxl6*,*7*,*8* mutant plants. As shown in Figure 2a–b, *smxl6*,*7*,*8* mutant plants could maintain higher RWC than WT at similar soil moisture contents during the soil-drying experiment. These lower water loss rates observed with *smxl6*,*7*,*8* mutant plants during drought might be attributed to several factors, such as decrease in stress-induced cell membrane damage. To verify this hypothesis, we compared the percentage of electrolyte leakages from *smxl6*,*7*,*8* mutant and WT during the soil-drying experiment. Our data revealed that *smxl6*,*7*,*8* mutant had lower electrolyte leakage rates than WT plants during water stress (Figure 2c), suggesting that loss-of-functions of the SMXL6, 7 and 8 proteins contributed to decreased drought-induced cell membrane damage of the *smxl6*,*7*,*8* mutant plants.

SLs have been reported to positively regulate anthocyanin biosynthesis in *Arabidopsis* plants [23]. We were then curious whether the anthocyanin content was altered in *smxl6*,*7*,*8* mutant plants, particularly under drought, in comparison with WT plants. We found that the anthocyanin contents were higher in *smxl6*,*7*,*8* mutant than WT plants under both well-watered and drought conditions (Figure 2d,e), with higher differential levels being observed under drought. This finding suggests the negative roles of SMXL6, 7 and 8 not only in basal anthocyanin biosynthesis but also drought-induced anthocyanin accumulation in *Arabidopsis* plants. As anthocyanins play a role in drought resistance by scavenging drought-induced ROS [31,32], increased anthocyanin accumulation in *smxl6*,*7*,*8* mutant plants under drought conditions is also an important adaptive mechanism for their enhanced drought resistance.

### 3.3. Arabidopsis smxl6,7,8 Mutant Plants Display Increased Leaf Surface Temperature and Reduced Cuticular Permeability

**Figure 3:**
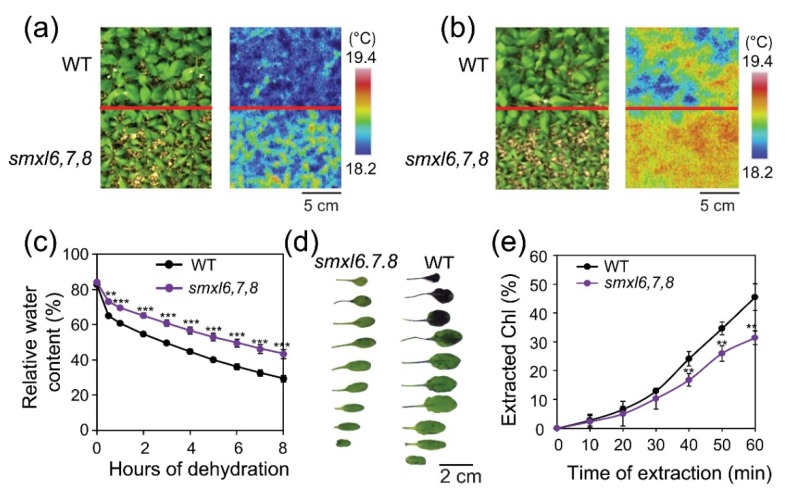
Leaf surface temperatures and cuticular permeability of *smxl6*,*7*,*8* mutant and wild-type (WT) plants. (**a**,**b**) Leaf surface temperatures of the two genotypes without (**a**) and with soil-drying for seven days (**b**). (**c**) Relative water contents of rosette leaves of the two genotypes under dehydration. (**d**) Detection of cuticular permeability by toluidine blue staining of rosette leaves of four-week-old WT and *smxl6*,*7*,*8* mutant plants grown under normal growth conditions. (**e**) Percentages of chlorophyll (Chl) leaching from the rosette leaves of four-week-old WT and *smxl6*,*7*,*8* mutant plants grown under normal growth conditions. Data shown in (**c**) and (**e**) are means and SEs (*n* = 5 plants). Asterisks show significant differences between the two genotypes at each time point (Student’s *t*-test; ** *p* < 0.01 and *** *p* < 0.001).

To gain further mechanistic insights into the roles of SMXL6, 7 and 8 proteins in increasing leaf water loss during drought, we compared the leaf temperatures and cuticular permeability of *smxl6*,*7*,*8* mutant and WT plants, because these traits infer stomatal water loss [33,34,35] and cuticular water loss [36], respectively. Consistent with their enhanced drought resistance and higher RWC observed under drought, *smxl6*,*7*,*8* mutant plants showed higher leaf surface temperatures than WT plants with or without soil-drying for 7 days (Figure 3a,b), which was also confirmed by the slower leaf water loss process observed with *smxl6*,*7*,*8* mutant plants in relation to WT under a dehydration treatment (Figure 3c). As cuticle is an important layer in the leaf surface to prevent water loss [36], we were curious whether the cuticular water permeability in leaves of the *smxl6*,*7*,*8* mutant was affected. Therefore, we carried out both TB staining and Chl leaching assays of rosette leaves from WT and *smxl6*,*7*,*8* mutant plants to assess their differential cuticular water permeability. We found that WT rosette leaves showed enhanced TB staining than *smxl6*,*7*,*8* mutant plants, especially their older leaves (Figure 3d). Additionally, we observed that Chls leached much faster from rosette leaves of WT than from that of s*mxl6*,*7*,*8* mutant plants (Figure 3e). These results collectively suggested that mutations in *SMXL6*, *7* and *8* genes reduced cuticular water permeability in the s*mxl6*,*7*,*8* mutant plants.

### 3.4. Arabidopsis smxl6,7,8 Mutant Plants Show Increased ABA Sensitivity

**Figure 4:**
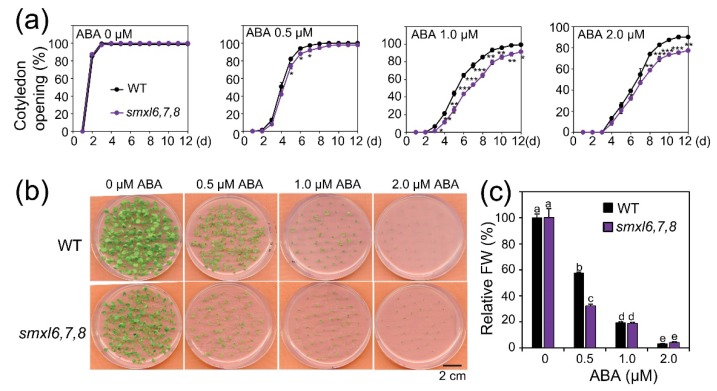
Abscisic acid (ABA) responsiveness of *smxl6*,*7*,*8* mutant and wild-type (WT) plants. (**a**) Cotyledon opening of the two genotypes in responses to different ABA concentrations. Data are means and standard deviations of three independent experiments (*n* = 3, 50 seeds/genotype/experiment). Asterisks show significant difference between the *smxl6*,*7*,*8* mutant and WT plants at each time point (Student’s *t*-test; * *p* < 0.05, ** *p* < 0.01 and *** *p* < 0.001). (**b**) Growth of the two genotypes on medium supplied with different ABA concentrations. Representative pictures of 14-day-old plants are shown. (**c**) Relative fresh weights (FW) of 14-day-old *smxl6*,*7*,*8* mutant and WT seedlings grown on medium supplied with different ABA concentrations. Data show means and standard errors (*n* = 5 replicates, six seedlings/replicate). Letters above the error bars show significant differences in all combinations (Tukey’s honest significant difference test; *p* < 0.05).

ABA responsiveness is an important trait associated with plant drought resistance [37,38]. We, therefore, examined whether the enhanced drought resistance of the *smxl6*,*7*,*8* mutant had any correlation with its ABA responsiveness. We used both cotyledon opening and growth inhibition assays to compare the ABA responsiveness of *smxl6*,*7*,*8* mutant and WT seedlings. Our results revealed that while *smxl6*,*7*,*8* mutant and WT seeds showed comparable cotyledon opening rates in the absence of ABA, *smxl6*,*7*,*8* mutant seeds showed lower percentages of cotyledon opening than WT seeds by addition of various concentrations of ABA to the medium (Figure 4a), suggesting that the *smxl6*,*7*,*8* mutant had higher ABA responsiveness than WT. This finding was also supported by the results of a growth inhibition assay (Figure 4b,c), in which the *smxl6*,*7*,*8* mutant plants exhibited higher FW reduction rate than WT plants in the presence of 0.5 µM ABA (Figure 4c). Our results collectively suggest that an enhanced ABA responsiveness might contribute to improved drought resistance of the *smxl6*,*7*,*8* mutant plants.

### 3.5. Arabidopsis smxl6,7,8 Mutant Plants Display Enhanced Oxidative Stress Resistance

**Figure 5:**
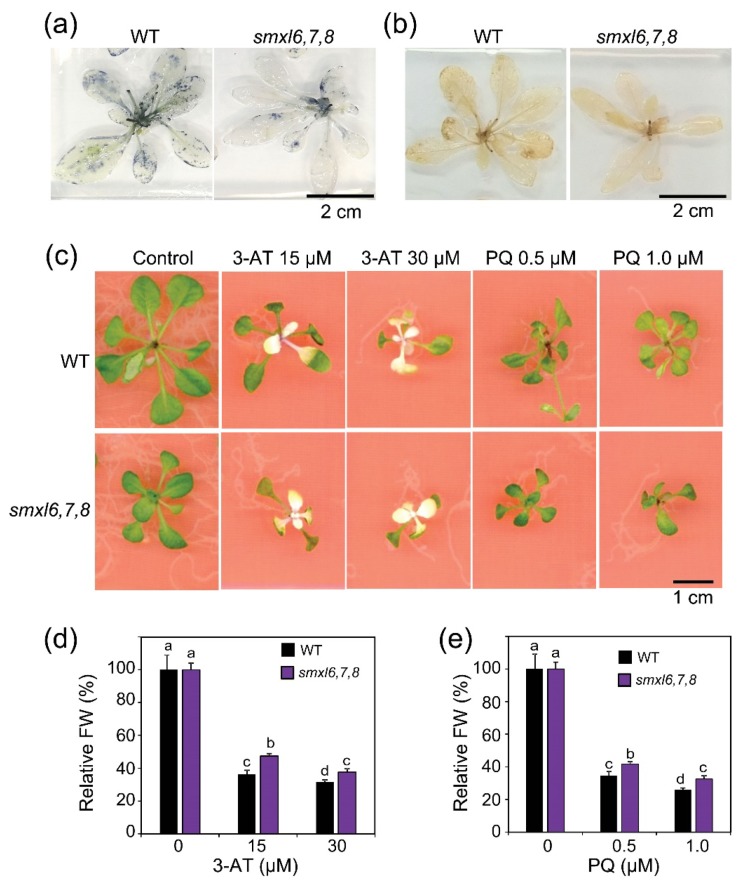
Detoxification capacity of reactive oxygen species in *smxl6*,*7*,*8* mutant and wild-type (WT) plants. (**a**,**b**) Histochemical analyses of O_2_**^.^**^–^ accumulation (**a**) and H_2_O_2_ accumulation (**b**) through nitro blue tetrazolium (**a**) and 3,3′-diaminobenzidine (**b**) stainings of rosette leaves of 28-day-old *smxl6*,*7*,*8* mutant and WT plants. (**c**) Growth inhibition assays of the two genotypes using 3-amino-1,2,4-triazole (3-AT) and paraquat (PQ). Representative pictures of 21-day-old plants are shown. (**d**,**e**) Relative fresh weights (FWs) of 21-day-old *smxl6*,*7*,*8* mutant and WT plants grown on medium supplied with different concentrations of 3-AT (**d**) or PQ (**e**). Data show means and standard errors (*n* = 10 plants/genotype). Letters above the error bars show significant differences in all combinations (Tukey’s honest significant difference test; *p* < 0.05).

Drought induces production of ROS, including O_2_**^.^**^–^ and H_2_O_2_ that cause oxidative damage; and thus, enhancement of ROS-scavenging activity is one of the important mechanisms in plant protection against water stress [31,39]. We hypothesized that ROS homeostasis might be affected in *smxl6*,*7*,*8* mutant plants; and thus, we investigated the accumulation of O_2_**^.^**^–^ and H_2_O_2_ using NBT and DAP stainings, respectively. Our data indicated that the rosette leaves of *smxl6*,*7*,*8* mutant plants produced lower levels of O_2_**^.^**^–^ and H_2_O_2_ than that of WT plants (Figure 5a,b), suggesting that *smxl6*,*7*,*8* mutant plants might possess higher ROS-scavenging capacity than WT plants. To confirm this hypothesis, we measured the responsiveness of WT and *smxl6*,*7*,*8* mutant plants to PQ and 3-AT treatments (Figure 5c), which could induce higher levels of ROS in leaves. Results revealed that *smxl6*,*7*,*8* mutant plants suffered lower levels of growth inhibition by PQ and 3-AT treatments than WT plants (Figure 5d,e), indicating that the *smxl6*,*7*,*8* mutant plants possessed higher ROS detoxification capacity than WT plants. Thus, this finding indicated that loss-of-functions of SMXL6, 7 and 8 proteins resulted in enhanced ROS detoxification capacity, which might help *smxl6*,*7*,*8* mutant plants survive oxidative stress when drought occurs.

### 3.6. Expression Analysis of Marker Genes

In the next line of our study, we examined whether the physiological and biochemical changes observed in the *smxl6*,*7*,*8* mutant plants were associated with the changes in expression levels of some genes involved in these processes. We first compared the transcript levels of several well-known genes involved in anthocyanin biosynthesis, cuticle formation, water transpiration, cellular dehydration and ABA responsiveness in *smxl6,7,8* mutant and WT plants. For example, *FLAVONOID 3′-HYDROXYLASE* (*F3′H*) and *WAX ESTER SYNTHASE/ACYL-COENZYME A:DIACYLGLYCEROL ACYLTRANSFERASE 1* (*WSD1*) are important genes involved in anthocyanin biosynthesis and cuticle formation, respectively [40,41], while *SENESCENCE-ASSOCIATED GENE 29* (*SAG29*) and *ABA INSENSITIVE 5* (*ABI5*) genes have been widely used as marker genes for ABA response [42,43]. Genes like *LATE EMBRYOGENESIS ABUNDANT 18* (*LEA18*), *LEA76* and *ABA-RESPONSE PROTEIN* (*ABR*) encoding LEA proteins, and *PROLINE DEHYDROGENASE 1* (*PDH1*) and *PDH2* encoding enzymes involved in proline catabolism have been reported to participate in regulating plant response to drought, as both LEAs and proline play important roles in protecting plants from cellular dehydration [44,45,46,47,48,49,50,51,52,53]. *WRKY46* and its downstream gene *QUA-QUINE STARCH* (*QQS*) have been known to be involved in controlling water transpiration by negatively regulating stomatal closure [54].

**Figure 6:**
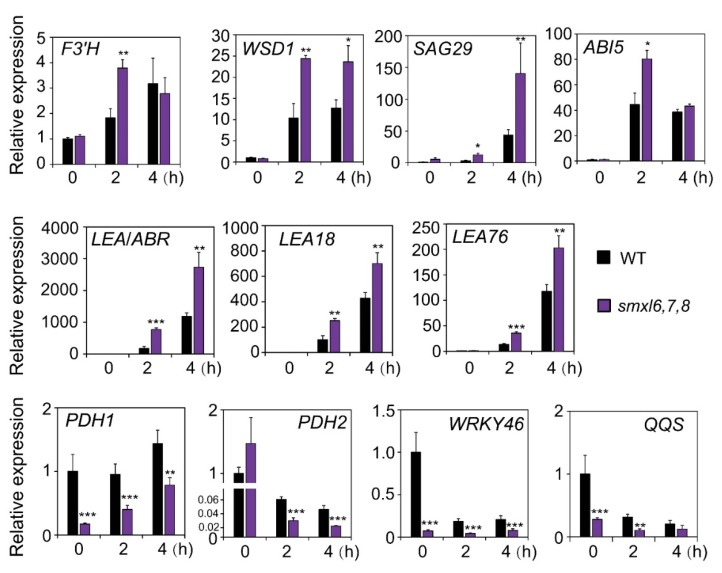
Expression patterns of several marker genes related to several drought resistance-associated traits in *smxl6*,*7*,*8* mutant and wild-type (WT) plants under normal and dehydration conditions. Rosette leaves of 24-day-old soil-grown plants were used for qRT-PCR analysis. Relative transcript levels were normalized to a value of 1 in the non-dehydrated WT. Data shown are means and standard errors (*n* = 3 biological replicates). Asterisks show significant differences between the *smxl6,7,8* mutant and WT plants in the same treatment condition (Student’s *t*-test; * *p* < 0.05; ** *p* < 0.01 and *** *p* < 0.001).

We recorded higher expression levels of *F3′H* gene after 2 h of dehydration, and of *WSD1*, *SAG29* and *ABI5* genes after 2 and 4 h of dehydration in *smxl6,7,8* mutant than WT plants (Figure 6), which showed a positive correlation with the improved anthocyanin biosynthesis (Figure 2d,e), reduced cuticle permeability (Figure 3d,e) and increased ABA responsiveness (Figure 4) of the *smxl6,7,8* mutant in relation to the WT (Figure 4). Furthermore, we observed higher transcript levels of *LEA*/*ABR*, *LEA18* and *LEA76*, while lower expression levels of *PDH1*, *PDH2*, *WRKY46* and *QQS*, genes, particularly during dehydration, in *smxl6,7,8* mutant than in WT plants (Figure 6), which might together contribute to protect plant from dehydration damage. These data suggested that loss-of-functions of the SMXL6, 7 and 8 proteins enhanced the examined drought-related traits in *smxl6,7,8* mutant plants by modulating the expression levels of at least these investigated marker genes.

## 4. Discussion

Previous investigations reported that *SMXL6*, *7* and *8* genes redundantly regulate shoot branching and leaf morphology as the members of SL signaling [6,7]. However, compelling evidence for the functions of *SMXL6*, *7* and *8* genes in other phenotypes controlled by SL signaling, such as leaf senescence, secondary growth and drought resistance [7,19,21], is still lacking. In the present study, by comparing drought resistance levels of various combinations of knock-out mutants of *SMXL6*, *7* and *8* genes, including single, double and triple mutants (Figure 1; Figure S1), we firmly showed that SMXL6, 7 and 8 are involved in regulating drought resistance in *Arabidopsis* plants as redundant negative regulators. Since SMXL6, 7 and 8 are repressors of the SL signaling [6,7], our results provide not only convincing proof for the functions of these three repressors but also an additional evidence to strengthen the positive role of SLs in regulating drought resistance in plants as reported earlier by numerous studies [16,17,18,19,20,21]. For instance, SL-depleted (e.g., *max3* and *max4*) and SL-receptor (e.g., *d14*) *Arabidopsis* mutant plants, and SL-depleted *L. japonicus* (e.g., *LjCCD7*-silenced) and tomato (e.g., *SlCCD7*-silenced) transgenic plants were shown to exhibit susceptible phenotypes to various water-deficit stress conditions [16,17,18,19,20,21]. Additionally, the differential level in drought resistance of *smxl6*,*7*,*8* mutant versus WT (6.2–8.6-fold differences in survival rate of *smxl6*,*7*,*8* mutant, compared with WT plants; Figure 1 and Figure S1) was much higher than the previously reported differential level in drought susceptibility of *d14* mutant versus WT (1.7–2.2-fold differences in survival rate of WT, compared with *d14* mutant plants) [19], suggesting that the SMXL6, 7 and 8 repressors might act in other pathway(s) through a yet-unknown ‘promiscuity’ in the interaction dynamics to regulate plant response to drought.

Consistent with their enhanced drought resistance, *smxl6*,*7*,*8* mutant plants showed ability to reduce leaf water loss and enhance cell membrane integrity under drought stress conditions (Figure 2b,c). These results suggested that *SMXL6, 7* and *8* genes were involved in the regulation of mechanisms associated with, at least, these investigated drought resistance-related traits. An increase in cell membrane integrity can help plants prevent leaf water loss as reported by various studies [55,56,57,58]. Furthermore, the higher leaf surface temperatures observed in *smxl6*,*7*,*8* mutant plants in comparison with WT plants under both well-watered and water-deficit conditions supported the reduced leaf water transpiration from the *smxl6*,*7*,*8* mutant versus WT plants (Figure 3a–c). Since a growing body of studies has revealed convincing evidence for the positive correlations between the leaf surface temperature and stomatal closure, leaf temperature assay has been widely used in indirect monitoring stomatal movement [33,34,35]. Thus, the increase in leaf surface temperatures of *smxl6*,*7*,*8* mutant plants suggests that loss-of-functions of *SMXL6, 7* and *8* genes result in enhanced SL signaling, which in turn might promote stomatal closure and consequently drought resistance. Indeed, increasing evidence has indicated the promoting roles of SLs and SL signaling in stomatal closing [15,16,18,19,20,21,59], further supporting the involvement of SMXL 6, 7 and 8 repressors in regulating stomatal movement through the SL signaling. In addition, various SL-deficient plant species have shown slower stomatal closing rates in the presence of ABA than WT plants [16,20,21], and reduced ABA sensitivity in stomatal response assays, compared with WT [20,21], suggesting the existence of an ABA-dependent mediation of stomatal closure by SLs and SL signaling. In the present study, we also found that the *smxl6*,*7*,*8* mutant plants were hypersensitive to ABA in both cotyledon opening and growth inhibition assays (Figure 4), which is in agreement with the positive roles of SLs and SL signaling in regulating ABA responsiveness [16,19,20,21]. An increase in ABA responsiveness may lead to activation of downstream signaling components, such as mitogen-activated protein kinase cascade module [60,61] and ABA–RESPONSIVE ELEMENT BINDING PROTEIN 1 pathway [62], resulting in enhanced drought resistance both dependently [61] and independently [62] of the status of stomatal closure.

Previously, reduced cuticle thickness and increased Chl leaching rates were recorded in the *max2* [15], *kai2* and *kai2 d14* [17,19], but not in the *d14* mutant plants [17,19]. Interestingly, *kai2 d14* showed significantly higher Chl leaching rates than *kai2* mutant plants [19]. These results suggest that KAR-specific KAI2 signaling, i.e., a yet-unknown endogenous KAI2 ligand (KL)-mediated signaling is involved in regulation of cuticular water permeability [17,19], which is an important drought resistance-related trait [36]. However, whether the SL-mediated signaling plays a role in regulating this trait, investigations of other members of the SL signaling is also required to obtain a firm conclusion. Accordingly, we investigated the *smxl6*,*7*,*8* mutant, and the results showed that the *smxl6*,*7*,*8* mutant plants had decreased cuticular permeability as indicated by both TB staining and Chl leaching assays (Figure 3d,e). This finding demonstrated that SL signaling was, indeed, also involved in cuticle formation, at least through the functions of the *SMXL6, 7* and *8* genes. It will be then interesting to investigate both cuticular permeability and cuticle thickness in the SL-depleted and SL-signaling mutants of different plant species in the future, which might open a new opportunity for development of improved drought-resistant crop cultivars by reducing cuticular water permeability.

Several studies have shown that anthocyanin contents were reduced in the SL-biosynthetic *max1* and *max2* mutant plants, and exogenous applications of *rac-*GR24 (widely used as a SL analog, but can be recognized by both D14 and KAI2) enhanced anthocyanin accumulations in WT and *max1* [23], and *d14* and *kai2* [63], but not in *max2* [23] mutant plants, suggesting that SLs positively regulate the production of anthocyanins in plants. In accordance with these observations, we found increased anthocyanin accumulations in *smxl6*,*7*,*8* mutant versus WT plants under both well-watered and drought stress conditions (Figure 2d,e), indicating that SLs control anthocyanin production in plants through all SL signaling members identified so far, namely D14, MAX2 and SMXL 6, 7 and 8. Anthocyanins have been well known for their antioxidant functions that can help plants to reduce oxidative damage caused by environmental stresses, including drought [32]. Thus, the increased levels of anthocyanins in *smxl6*,*7*,*8* mutant plants might enhance their antioxidant defense as indicated by the decreases in O_2_**^.^**^–^ and H_2_O_2_ contents in the mutant versus WT plants (Figure 5a,b). Accordingly, the *smxl6*,*7*,*8* mutant plants showed enhanced oxidative stress resistance as evidenced by their lower growth inhibition rates on the medium containing either PQ or 3-AT in comparison with WT plants (Figure 5d,e).

It was then interesting to provide molecular insights, at least at the expression levels, into candidate genes potentially regulated by the SMXL6, 7 and 8. We then selected several marker genes, which are related to anthocyanin biosynthesis (*F3′H*), cuticular water permeability (*WSD1*), ABA responsiveness (*SAG29* and *ABI5*), control of water transpiration (*WRKY46* and *QQS*) and prevention of cellular dehydration (*LEA* genes, and *PDH1* and *PDH2* genes), for comparison of their expression levels in the *smxl6*,*7*,*8* mutant and WT plants (Figure 6). Our expression data revealed a good correlation between the transcript levels of the tested genes and the enhancement in investigated drought tolerance-related traits observed with *smxl6*,*7*,*8* mutant versus WT plants (Figure 2, Figure 3, Figure 4, Figure 5 and Figure 6). These results suggested that SMXL6, 7 and 8 negatively regulate the expression of some genes, such as the *F3′H*, *WSD1*, *SAG29*, *ABI5* and *LEA* genes (Figure 6), involved in improvement of drought-related physiological and biochemical traits like anthocyanin contents, antioxidant properties, cell membrane integrity, cuticle formation, as well as ABA responsiveness, thereby affecting plant response to drought (Figure 2, Figure 4 and Figure 5) [19,32,53]. Additionally, down-regulation of proline catabolism-related genes, such as *PDH1* and *PDH2* [46,47], recorded in *smxl6*,*7*,*8* mutant versus WT plants (Figure 6) suggested that the catabolism process of proline was weaker in this mutant than in WT plants, which would help maintain appropriate levels of proline in the *smxl6*,*7*,*8* mutant for better protection against osmotic stress damage. Investigations of changes in proline homeostasis in various SL-signaling and KAR-signaling mutants in a comparative manner would be an interesting future study. Furthermore, it was reported earlier that *WRKY46* and its direct target *QQS* positively regulates the stomatal opening and stomatal conductance via modulating starch degradation to increase malate ion (C_4_H_4_O_5_^2−^) accumulation in guard cells [54]. Thus, the observed down-regulation of *WRKY46* and *QQS* genes in *smxl6*,*7*,*8* mutant plants, compared with WT (Figure 6), might induce stomatal closing, as supported by the observed increase in leaf surface temperature in these mutant plants versus WT (Figure 3a–c), thereby helping *smxl6*,*7*,*8* mutant plants to reduce water loss to survive drought conditions (Figure 1). It is also important to note that the expression levels of *SMXL6*, *7* and *8* genes were down-regulated by early dehydration (Figure S2), suggesting a possible mechanism in which drought triggers down-regulation of these genes as an adaptive means to survive under adverse drought conditions.

It should be noted that the higher leaf surface temperature of the *smxl6*,*7*,*8* mutant than the WT plants under normal growth conditions (Figure 3a) might infer that the *smxl6*,*7*,*8* mutant plants would have narrower stomatal aperture size even under non-stressed conditions, as also suggested by down-regulation of *WRKY46* and *QQS* genes in well-hydrated *smxl6*,*7*,*8* mutant plants, compared with WT (Figure 6). In support of this idea, several reports have shown that under normal growth conditions, in comparison with WT plants, (i) the *Arabidopsis* SL-biosynthetic *max1*, *max3* and *max4*, and SL-signaling *max2* and *d14* mutant plants exhibited larger stomatal aperture size [19,59], and (ii) *d14* mutant plants displayed lower leaf surface temperature [19]. In the future, it will be very interesting to compare the stomatal aperture size, stomatal density, stomatal conductance and water use efficiency of *smxl6*,*7*,*8* mutant, in comparison with WT and perhaps with the KL signaling-related *smax1 smxl2* double mutant [64], in detail for in-depth understanding of the SMAX1/SMXL protein-mediated mechanisms underlying water evaporation prior to exploring the homologs of these genes for the development of crop cultivars with improved drought resistance by gene editing.

## 5. Conclusions

Our results demonstrated that SMXL6, 7 and 8 proteins negatively regulated drought resistance mainly through their actions in (i) enhancing both stomatal and non-stomatal water transpiration as indicated by leaf temperature and cuticular permeability assays, respectively, (ii) decreasing ABA sensitivity and cell membrane integrity, (iii) reducing antioxidant defense by at least repressing anthocyanin production and (iv) upregulating *LEA* genes to better protect the cells from dehydration damage. The findings of this study opens new avenues for future research and application.

## Supplementary Materials

The following are available online at https://www.mdpi.com/2218-273X/10/4/607/s1, Figure S1. Drought-resistant phenotypes of various smxl single, double and triple mutant plants, Figure S2. Expression of SMXL6, 7 and 8 genes in wild-type plants under normal and dehydration conditions, Table S1: Primers used for qRT-PCR.
