# Supplementary material for: Negative Roles of Strigolactone-Related SMXL6, 7 and 8 Proteins in Drought Resistance in Arabidopsis
Source: Biomolecules. 2020 Apr 14;10(4):607. doi: 10.3390/biom10040607 (PMC7226073; doi:10.3390/biom10040607)
Supplement: Supplementary file 1 [file biomolecules-10-00607-s001.pdf]

Supplementary data

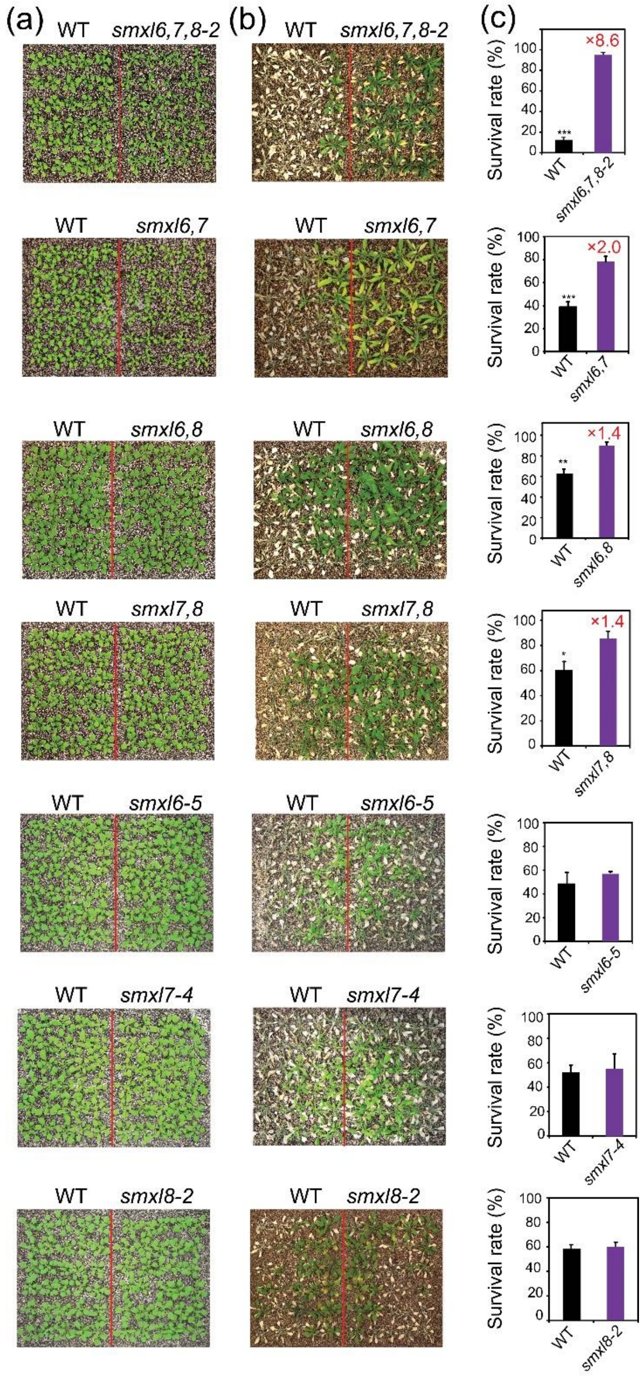

Figure S1. Drought-resistant phenotypes of various *smxl* single, double and triple mutant plants. Comparisons of different mutant and wild-type (WT) plants were performed using the 'same tray' method. (a) WT and different mutant plants were grown in pairs for 21 days under well-watered conditions in a tray. (b) Water was withheld until distinguishable differences were observed between the two genotypes, then re-watering was conducted. Pictures were taken five days after re-watering, and after inflorescences were removed. (c) Means and standard errors of three independent experiments (n = 3, 30 plants/genotype/experiment) were used to estimate the survival rates of investigated WT and

different mutant plants. Asterisks indicate significant difference between the two genotypes (Student's *t*-test; $*p < 0.05$, $**p < 0.01$, $***p < 0.001$). Red numbers above the error bar indicate the fold-change in survival rate between the two genotypes.

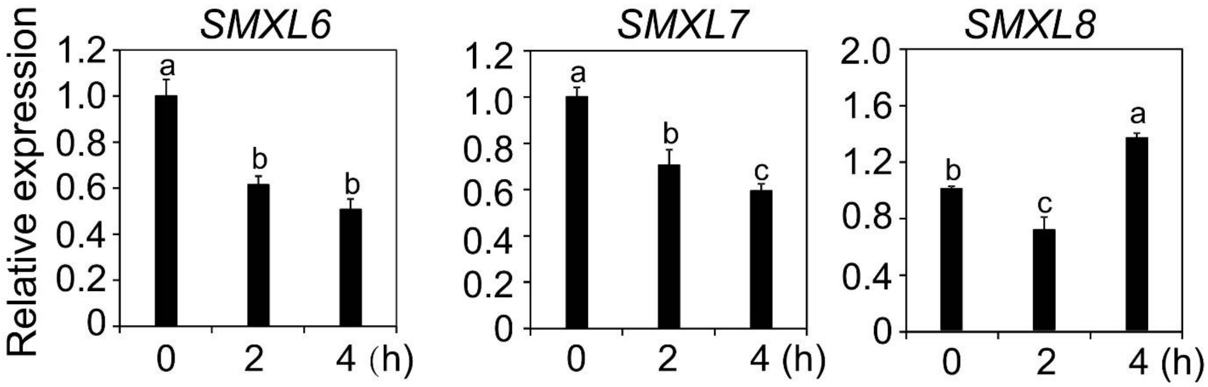

Figure S2. Expression of *SMXL6, 7* and *8* genes in wild-type plants under normal and dehydration conditions. Rosette leaves of 24-day-old soil-grown plants were used for qRT-PCR analysis. Relative transcript levels were normalized to a value of 1 in the non-dehydrated sample. Data are means and standard errors ($n = 3$ biological replicates). Letters above the error bars show significant differences in all combinations (Tukey's honest significant difference test; $p < 0.05$).

Table S1. Primers used for qRT-PCR

**Gene name**	**AGI code**	**Prime name**	**Primer pair (5'-3' sequence)**
F3'H	AT5G07990	F3'H-F	AGACATCGCTCAGCTTCCTT
		F3'H-R	TGGTGGATGAAGCCTGAAAT
WSD1	AT5G37300	WSD1-F	GCTTGGTGGTTGTTTGTTGG
		WSD1-R2	TCGGGTTACCCATAAGAGGG
SAG29	AT5G13170	SAG29-F	GCCACCAGGGAGAAAAGG
		SAG29-R	CCACGAAATGTGTTACCATTAGAA
ABI5	AT2G36270	ABI5-F	GCCACCGGTTTTTAGACACA
		ABI5-R	AGTAAACGGATGATTCTCACCAC
PDH1	AT3G30775	PDH1-F	AGCTGCCAAATCTTTACCAACATC
		PDH1-R	GCTTCCATGAGAGTTTGAAGTTCG
PDH2	AT5G38710	PDH2-F	GTGCTTTAGACCGCCAACTT
		PDH2-R	TGTGTCTTCACCAAGCCATAA
WRKY46	AT2G46400	WRKY46-F	ACATCACATCCCCGAAGACG
		WRKY46-R	ACTTCTTCGGACTTGGTCGG
QQS	AT3G30720	QQS-F	TTCTCCACAGCGACCAGTTG
		QQS-R	CCCTCATTTTGAGCCTTGCG
LEA/ABR	AT3G02480	LEA/ABR-F	AGACTCCTTGCAACAGACTGGAC
		LEA/ABR-R	TCCTTGACGACATCAGCAGCTC
LEA18	AT2G35300	LEA18-F	GGTCATGCGGAGAAGACGAT
		LEA18-R	GCATGCTCAGCCTTGGATTG
LEA76	AT3G15670	LEA670-F	TTATCGGAGACAGGCGAAGC
		LEA670-R	CACCCGTCTTGTCTCTACCG
SMXL6	AT1G07200	SMXL6-F	CGGGCTATTGAGACCAAAGA
		SMXL6-R	GGACTCACCGACTGAAAACC
SMXL7	AT2G29970	SMXL7-F	AGCCCTTAAGTCTCAGCGTTCG
		SMXL7-R	TACGCTTCGTCTTCGTTTGCTTC
SMXL8	AT2G40130	SMXL8-F	GCCACCTTGGTTACAAATGACCAC
		SMXL8-R	ACCCTTCTTTGGTCTGCACTTGG
UBQ10	AT4G05320	UBQ10-F	GAAGTTCAATGTTTCGTTTCATGT
		UBQ10-R	GGATTATACAAGGCCCCAAAA
